# Development and validation of nomograms integrating immune‐related genomic signatures with clinicopathologic features to improve prognosis and predictive value of triple‐negative breast cancer: A gene expression‐based retrospective study

**DOI:** 10.1002/cam4.1880

**Published:** 2019-01-24

**Authors:** Kang Wang, Hai‐Lin Li, Yong‐Fu Xiong, Yang Shi, Zhu‐Yue Li, Jie Li, Xiang Zhang, Hong‐Yuan Li

**Affiliations:** ^1^ Department of the Endocrine and Breast Surgery The First Affiliated Hospital of Chongqing Medical University, Chongqing Medical University Chongqing China; ^2^ Department of Gastrointestinal Surgery The First Affiliated Hospital of Chongqing Medical University, Chongqing Medical University Chongqing China; ^3^ Division of Biostatistics and Data Science, Department of Population Health Sciences Medical College of Georgia, Augusta University Augusta Georgia; ^4^ West China School of Public Health Sichuan University Chengdu China; ^5^ Institute of Hospital Management West China Hospital, Sichuan University Chengdu China; ^6^ West China Hospital/West China School of Nursing Sichuan University Chengdu China

**Keywords:** immune‐related genomic signatures, nomogram, triple‐negative breast

## Abstract

**Purpose:**

Accumulating evidence indicated that triple‐negative breast cancer (TNBC) can stimulate stronger immune responses than other subtypes of breast cancer. We hypothesized that integrating immune‐related genomic signatures with clinicopathologic factors may yield a predictive accuracy exceeding that of the currently available system.

**Methods:**

Ten signatures that reflect specific immunogenic or immune microenvironmental features of TNBC were identified and re‐analyzed using bioinformatic methods. Then, clinically annotated TNBC (n = 711) with the corresponding expression profiles, which predicted a patient's probability of disease‐free survival (DFS) and overall survival (OS), was pooled to evaluate their prognostic values and establish a clinicopathologic‐genomic nomogram. Three and two immune features were, respectively, selected out of 10 immune features to construct nomogram for DFS and OS prediction based on multivariate backward stepwise Cox regression analyses.

**Results:**

By integrating the above immune expression signatures with prognostic clinicopathologic features, clinicopathologic‐genomic nomograms were cautiously constructed, which showed reasonable prediction accuracies (DFS: HR, 1.79; 95% CI, 1.46‐2.18, *P* < 0.001; AUC, 0.71; OS: HR, 1.96; 95% CI, 1.54‐2.49; *P* < 0.001; AUC, 0.73). The nomogram showed low‐risk subgroup had higher immune checkpoint molecules (PD‐L1, PD‐1, CTLA‐4, LAG‐3) expression and benefited from radiotherapy (HR, 0.2, 95% CI, 0.05‐0.89; *P* = 0.034) rather than chemotherapy (HR, 1.26, 95% CI, 0.66‐2.43; *P* = 0.485).

**Conclusions:**

These findings offer evidence that immune‐related genomic data provide independent and complementary prognostic information for TNBC, and the nomogram might be a practical predictive tool to identify TNBC patients who would benefit from chemotherapy, radiotherapy, and upcoming popularity of immunotherapy.

## 1 INTRODUCTION

As the most common female cancer, breast cancer is diagnosed in more than 266 120 patients in the United States each year.^1^ Although representing only about 10%‐20% of patients, triple‐negative breast cancer (TNBC) patients had much worse prognosis than other subtypes owing to an inherently aggressive clinical behavior and a lack of recognized molecular targets for therapy (ie, estrogen receptor (ER), progesterone receptor (PR), human epidermal growth factor receptor 2 (HER2)).^1^, ^2^

Besides generally documented clinicopathologic risk factors like tumor size, nodal status, and grade, no prognostic and predictive biomarkers are suitable for predicting those high‐risk patients with TNBC. Accumulating evidence indicated that TNBC can stimulate stronger immune responses than other subtypes of breast cancer,^3^ due to its higher genomic instability.^4^ It was well established that the immune system can help to reduce the risk of tumor spreading and maintain tumor dormancy,^2^, ^5^, ^6^ and tumor‐infiltrating lymphocytes (TILs) are focus of this area. Although subtypes of TILs had different impacts on the prognosis of TNBC, some TIL subtypes that were known to downregulate the immune paradoxically inferred a better prognosis in TNBC.^7^ Of note, the interobserver variance is still deemed too great to fulfill the application of TILs evaluation in a clinical setting.^8^, ^9^ It is therefore essential to investigate the specialized functions with regard to different subtypes of TILs.

Meanwhile, besides the microenvironment, we should not neglect importance of spatial context, those intra‐tumoral immune processes also correlated with cancer prognosis.^10^ As genomics has developed rapidly with the application of next‐generation sequencing technology in recent years, several elegant immune‐related signatures were proposed to predict the prognosis of cancers, such as macrophages/monocytes,^11^ overall lymphocyte infiltration,^12^ IFN‐g response,^13^ and wound healing signature.^14^ Moreover, immune expression signatures reflecting intra‐tumoral immune states were also constructed for TNBC.^15^, ^16^, ^17^, ^18^, ^19^, ^20^, ^21^, ^22^, ^23^ Rody et al defined the metagene that described a ratio of high B‐cell presence and low IL‐8 activity as a powerful new prognostic marker for TNBC. Another 95‐genes STAT1‐related immune metagene was constructed,^17^ and only this immune response module was associated with prognosis in the ER‐/HER2‐ subgroup. Additionally, an 8‐gene follicular T‐helper cells (Tfh) signature, signifying organized antitumor immunity, robustly predicted survival or preoperative response to chemotherapy in TNBC.^18^ Actually, those signatures may reflect specific immunogenic or immune microenvironmental features of TNBC that can determine the tumor phenotype, but these published signatures clearly showed low prediction accuracies and limited clinical application. Screening and integrating those immune‐related genomic signatures maybe rendered the insight into the heterogeneous biology of TNBC and may become useful for improved selection of patients who need additional treatment with new drugs targeting the immune system.

Cytotoxic chemotherapy is the mainstay of treatment option for patients with early or those with advanced TNBC, and TNBC is highly sensitive to chemotherapy with pathological complete remission (pCR) rating (30%‐40%) compared with luminal breast cancers (10%‐25%), respectively, after neoadjuvant chemotherapy.^5^, ^24^ Previous studies indicated that immune microenvironment collaborated with the action of chemotherapy in TNBC.^25^, ^26^ A large amount of studies has proved that high number of TILs was a significant predictor of survival outcomes, and increased in TILs was associated with a reduction in recurrence and death and increment in pCR rate to neoadjuvant chemotherapy.^6^, ^7^, ^8^, ^9^ Nevertheless, the triple‐negative paradox^20^ indicated that TNBC had high risk of recurrence without any treatment but also higher likelihood of benefit from treatment, likely due to inappropriate regimens or candidates for chemotherapy. Additionally, breast cancer patients with stage T3 or T4 disease or positive nodes would routinely receive radiotherapy to the chest wall or axillary bed.^27^ Interestingly, recent studies revealed that radiotherapy of the bulk tumor can also activate antitumor immune responses by re‐educating the immune system to recognize and reject cancer, converting the tumor into an in situ vaccine and exposing a wealth of previously hidden tumor‐associated antigens.^28^, ^29^, ^30^, ^31^ Of note is that TIL infiltration of tumor tissues could also predict the outcome of prostate cancer patients undergone salvage radiotherapy.^32^ Combining radiotherapy and immunotherapy can be quite potent, including against very large tumors,^33^ and further investigations are also ongoing in several cancers.^31^, ^34^, ^35^, ^36^

Integrating multiple biomarkers into a single model could substantially improve the prognostic value compared with that of a single model.^37^, ^38^ In this study, we applied the CIBERSORT^39^ to screen 22 TILs phenotypes and identify the most relevant prognostic immune cells in TNBC. Meanwhile, a systematic approach was conducted to evaluate the clinical usefulness of immune‐related genomic signatures in TNBC and then construct a composite nomograms (models) integrating immune‐related genomic signatures with clinicopathologic features in a training set. Furthermore, using another independent set, the capacity of the nomogram to stratify TNBC patients most likely to benefit from adjuvant regimens was further validated. We hypothesized that final composite nomograms would yield predictive accuracies exceeding that of the currently available prognostic system, and identification of patients stratified by nomograms may help the search for individualized regimens.

## 2 METHODS

### 2.1 Gene expression datasets

We identified gene expression data arrayed using Affymetrix Human Genome U133A or U133A plus 2.0 with clinically annotated data, Gene Expression Omnibus (GEO), ArrayExpress, The Cancer Genome Atlas (TCGA, 2016) and Molecular Taxonomy of Breast Cancer International Consortium (METABRIC, 2016) were systematically searched. All samples with gene expression data and survival data from breast carcinomas were eligible. Exclusion criteria included male breast cancer, primary metastasis breast cancer, normal tissue of breast cancer patients, and replicated cases. All of those studies previously were approved by their respective institutional review boards.

### 2.2 Data processing

Eleven gene expression datasets were available from public databases. For GEO datasets, the raw CEL files were normalized through a MAS5 algorithm and were mapped to Entrez GeneID using RefSeq and Entrez.^40^ When multiple probes mapped to the same GeneID, we used probes with the largest interquartile range across the samples. To convert count data to values more similar to those resulting from microarrays, the RNA sequencing data (TCGA and METABRC datasets) had been transformed using “voom” R package^41^ (variance modeling at the observational level). Batch effects were also recognized by fitting each gene to a linear model with 11 fixed effects for each dataset,^42^ including nine microarray datasets (GEO databases) and two RNA‐Seq datasets (TCGA and METABRAC databases).

### 2.3 Identification of triple‐negative breast cancer

The Affymetrix probes 205225_at, 208305_at, and 216836_s_at were chosen to represent ER, PR, and HER2 expression, respectively.^43^ We inferred receptor status using a 2‐component Gaussian mixture distribution model and parameters were estimated by maximum‐likelihood optimization as previously described.^44^ We calculated the posterior probability of negative expression status for ER, PR, and HER2, and then classified each sample as negative expression if its posterior probability was less than 0.5, which were carried out separately on a per‐dataset (GEO, TCGA and METABRIC) basis.

### 2.4 Identification of immune‐related genomic signatures with potential to predict prognosis

We searched literature database with the terms “triple‐negative breast cancer,” “TNBC,” “immune microenvironment,” “tumor‐infiltrating lymphocyte,” “TIL,” “survival,” “relapse,” “recurrence,” “prognostic,” and “prognosis” to identify gene expression studies on the predictive value of immune‐related functions in TNBC. The probesets or genes of those signatures were re‐annotated using SOURCE web tool (https://source-search.princeton.edu) to find the retired gene symbols or different names in each platform. The re‐annotated genes were then subjected to biological function enrichment analysis, and the online analytical tool DAVID (Database for Annotation, Visualization and Integrated Discovery^45^) was used to enrich gene ontology (GO) functions and Kyoto Encyclopedia of Genes and Genomes (KEGG) pathways. GO terms and KEGG pathways with significant enrichment false discovery rate (FDR) values less than 0.05 were selected for further analysis.

### 2.5 Statistical analysis

#### 2.5.1 Subclass prediction of TNBC patients

To characterize intra‐tumoral immune states, the preprocessed microarray (RNA‐seq) datasets along with templets that are prognostic immune‐related genomic signatures were used to classify each sample using the nearest template prediction (NTP) method^46^ as implemented in Gene Pattern software (Broad Institute of Harvard and MIT, Boston, MA). The (NTP) method provides a convenient way to make class prediction with assessment of prediction confidence computed in each single patient's gene expression data using only a list of signature genes and a test dataset. The significance of this classification was determined by a nominal p‐value estimated based on a null distribution for the distance generated through bootstrapping 1000 times, and generated FDR was used to correct the prior *P*‐values. The samples with FDR ≥ 0.05 were defined as uncertain gene signature presence (uncertainty), and in the contrast, residual samples (FDR < 0.05) were divided into the presence (good) or absence (poor) of gene signature where depended. The procedure started separately for each dataset and signature. After constructing prediction of presence, absence, uncertainty of signature as characteristics of each samples, we also investigated the concordance among these signature‐related characteristics according to Cramer's *V* coefficient of the paired prediction overlap.^47^

We inferred the relative proportions of 22 types of TILs using the Cell type Identification By Estimating Relative Subsets Of known RNA Transcripts (CIBERSORT) algorithm (https://cibersort.stanford.edu/),^39^ including B cells, T cells, natural killer cells, macrophages, dendritic cells, eosinophils, and neutrophils. Briefly, CIBERSORT was a deconvolution algorithm that used a signature matrix of 547 genes considered a minimal representation for each cell type and, based on those values, infers cell type proportions in data from bulk tumor samples. CIBERSORT generated a *P*‐value for the deconvolution for each sample after Monte Carlo sampling, which provided a measure of confidence in the results.

#### 2.5.2 Development, comparison, and validation of prognostic models

We divided all of samples into training and validation cohort according to dataset for assessing the predictors and outcomes. The distribution of clinicopathological variables between training and validation group was evaluated using the Pearson chi‐square test for categorical variables and Mann‐Whitney *U* test for continuous variables. Overall survival (OS) was calculated from the date of diagnosis or surgery to the date of death or last follow‐up, and disease‐free survival (DFS) was defined as the date of the diagnosis to the locoregional or distant recurrence or death from breast cancer, other cancer or other disease, whichever came first. OS as well as DFS were considered as censored status if patients were alive until date of last contact.

First, we assessed associations between inferred status of immune‐related genomic signature as well as proportions of immune cell types and survival outcomes using univariable Cox proportion hazard regression in training cohort, respectively. Then, the predictive effect of signatures‐related characteristics identified before was further analyzed based on multivariate backward stepwise Cox regression analyses, and final variables with obviously statistical significance (*P* < 0.05) would enter into our model (nomogram). After that, an individualized nomogram was constructed based on those known prognostic clinicopathological variables (age, stage) combined with genomic factors. Histologically, since majority of TNBC is grade III or poorly differentiated,^48^, ^49^ we excluded nuclear grade in the final model. Multiple imputation was used to impute those missing clinical data. To validate this model internally through 1000 bootstrap resamples, concordance index (C‐index) was calculated for the evaluation of the performance of predicting and discrimination ability by testing concordance between predicted probability and actual outcome. The samples were divided into three risk groups (high, intermediate, and low) according to the tertiles of the total scores calculated by the established nomogram in the training set. Last, external validation was conducted by assessing survival difference in three groups (high, intermediate, and low) defined by training nomogram. Kaplan‐Meier method was employed to visualize the survival distribution and conducted log‐rank tests to assess the differences between different risk groups and subgroups.

Analyses complied with STROBE criteria.^50^ All *P* values reported are two‐sided, which less than 0.05 were considered statistically significant. All analyses were conducted using R software (version 3.4.1).

## 3 RESULTS

### 3.1 Identification of eligible samples and immune‐related genomic signatures

**Figure 1:**
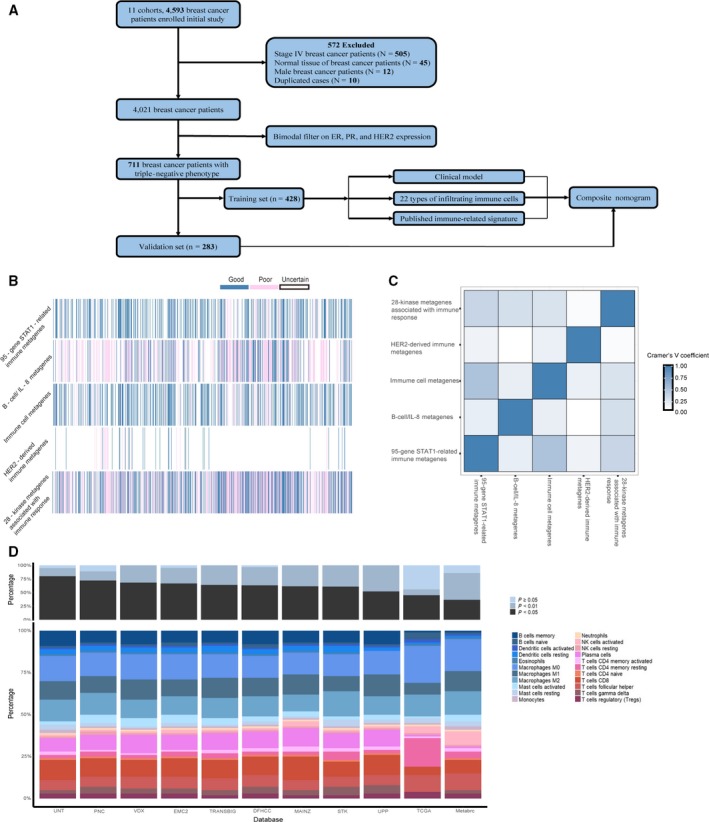
Immune‐related genomic landscape of TNBC based on gene expression profiling. A, Flowchart of the study design. B, Concordance of signature‐based prediction results. Each column represents the prediction of each individual sample. The blue, pink, and white bars indicate presence (good), absence (poor), and uncertain (uncertainty) prognoses of the corresponding signature, respectively. C, Heatmap of Cramer V coefficients showing correlation between these immune‐related genomic signatures. D, Bar charts summarizing immune cell subset proportions and CIBERSORT *P*‐value by study

We included 711 eligible samples from 11 independent cohorts including 4593 breast cancer patients with follow‐up data, and 3882 samples were excluded due to male breast cancer, stage IV breast cancer, normal breast samples, and subtypes other than TNBC (Figure 1A and Table S1). Then, we randomly divided the included samples into training set and validation set, and no significant differences in clinicopathologic characteristics between two cohorts were observed (all *P* > 0.05) (Table 1). Last, we addressed the possibility of selection bias due to missing data in multivariable models by deriving estimates based on multiply imputed datasets (10 cycles).

**Table 1:** Characteristics of patients and tumors included in this study (n = 711)

Characteristic	Training set (%)	Validation set (%)	*P* *
	N = 428	N = 283	
Age at diagnosis (y)†	55.6 ± 13.5	53.4 ± 12.7	0.01^a^
<40	50 (11.7)	35 (12.4)	
40‐50	88 (20.6)	78 (27.6)	
50‐60	101 (23.6)	64 (22.6)	
60‐70	83 (19.4)	53 (18.7)	
70+	62 (14.5)	25 (8.8)	
NA	44 (10.3)	28 (9.9)	
Grade			
I/II	47 (11.0)	29 (10.2)	0.94
III	209 (48.8)	141 (49.8)	
NA	172 (40.2)	113 (39.9)	
Lymph node metastasis			
No	214 (50.0)	135 (47.7)	0.77
Yes	71 (16.6)	46 (16.3)	
NA	143 (33.4)	102 (36.0)	
Tumor size (cm)			
<2	157 (36.7)	91 (32.2)	0.65
2‐5	190 (44.4)	133 (47.0)	
>5	22 (5.1)	15 (5.3)	
NA	59 (13.8)	44 (15.5)	
Stage			
I	51 (11.9)	29 (10.2)	0.77
II	144 (336.6)	89 (31.4)	
III	31 (7.2)	22 (7.8)	
NA	202 (47.2)	143 (50.5)	
Chemotherapy			
No	192 (44.9)	127 (44.9)	0.42
Yes	213 (49.8)	134 (47.3)	
NA	23 (5.4)	22 (7.8)	
Radiotherapy			
No	125 (29.2)	75 (26.5)	0.43
Yes	51 (11.9)	28 (9.9)	
NA	252 (58.9)	180 (63.6)	
Disease recurrence/metastasis			
No	283 (66.1)	187 (66.1)	0.22
Yes	145 (33.9)	94 (33.2)	
NA	0 (0.0)	2 (0.7)	
Overall death			
No	187 (43.7)	124 (43.8)	0.96
Yes	101 (23.6)	69 (24.4)	
NA	140 (32.7)	90 (31.8)	

NA, not available. ^†^Values are mean (SD). ^*^Pearson chi‐square test, except ^a^Mann‐Whitney *U* test.

To ascertain the clinically useful immune‐related genomic signature for TNBC, we comprehensively retrieved all of the published immune‐related genomic signatures of TNBC. As a result, nine immune‐related signatures^15^, ^16^, ^17^, ^18^, ^19^, ^20^, ^21^, ^22^, ^23^ of TNBC were published as valid prediction tools, four of which^15^, ^16^, ^18^, ^23^ did not report the prognostic value of containing gene were excluded (Table S2).

### 3.2 Prognostic performance of the published immune‐related signatures

To identify the correlations of immune expression signatures and TNBC patients' outcomes, the prognostic performances of the five signatures were evaluated in the training set using a modified NTP method as previously described. Five signatures were able to confidently stratify patients (FDR < 0.05) into good (presence) and poor (absence) subgroups, which were shown as Table 2 and Figure 1B. The signature “28‐kinase metagenes associated with immune response” was the most prevalent prediction in the whole set (77.0%), whereas signature “HER2‐derived prognostic predictor enriched in immune genes” was securely identified in only 6.0% TNBC patients. We sought to evaluate the concordance of these five signatures using Cramer's *V* coefficient, but no substantial association among the five signatures was found (Figure 1C).

**Table 2:** Immune‐related genomic signatures included in the study

Signature/author/year	Outcome	Number of genes in signature		Patients with the signature classified as^a^			Reference
		Original	Available (%)	Poor (%)	Uncertainty (%)	Good (%)	
95‐gene STAT1‐related immune metagenes; Desmedt et al; 2008.	NA	95	69 (72.6)	48 (6.8)	411 (57.8)	252 (35.4)	17
B‐cell/IL‐8 metagenes; Rody et al; 2011.	DFS	216	186 (86.1)	205 (28.8)	409 (57.5)	97 (13.7)	20
Immune cells metagenes; Nagalla et al; 2013.	DMFS	70	52 (74.3)	61 (8.6)	393 (55.3)	257 (36.1)	19
HER2‐derived prognostic predictor enriched in immune genes; Staaf et al; 2010	OS DMFS	14	13 (92.9)	15 (2.1)	668 (94.0)	28 (3.9)	22
28‐kinase metagenes associated with immune response; Sabatier et al; 2011.	DFS	368	275 (74.7)	286 (40.2)	163 (22.9)	262 (36.8)	21

DFS, disease‐free survival; DMFS, distant metastasis‐free survival; OS: overall survival.

a Samples were classified as presence, absence, or uncertainty by respective published genomic signatures based on prediction result (false discover rate [FDR] < 0.05) of nearest template prediction (NTP).

Figure 1D showed that the empirical CIBERSORT *P*‐value among 11 cohorts was generally consistent, with over half of the samples (n = 596) with a CIBERSORT *P* < 0.05 at 84% representation but none at 16% representation. Relative proportion of 22 TILs subsets showed relatively small differences across independent cohorts (Figure 1D). The most common immune cells were macrophages M0, macrophages M2, and T cells follicular helper with mean fraction of 18.7%, 13.0%, 9.3%, respectively. Patient samples were divided into three groups (low, medium, and high) according to the tertiles values of 22 immune cells fractions inferred in the training set, which were then applied to the validation set.

### 3.3 Independent prognostic factors for survival in the training set

**Figure 2:**
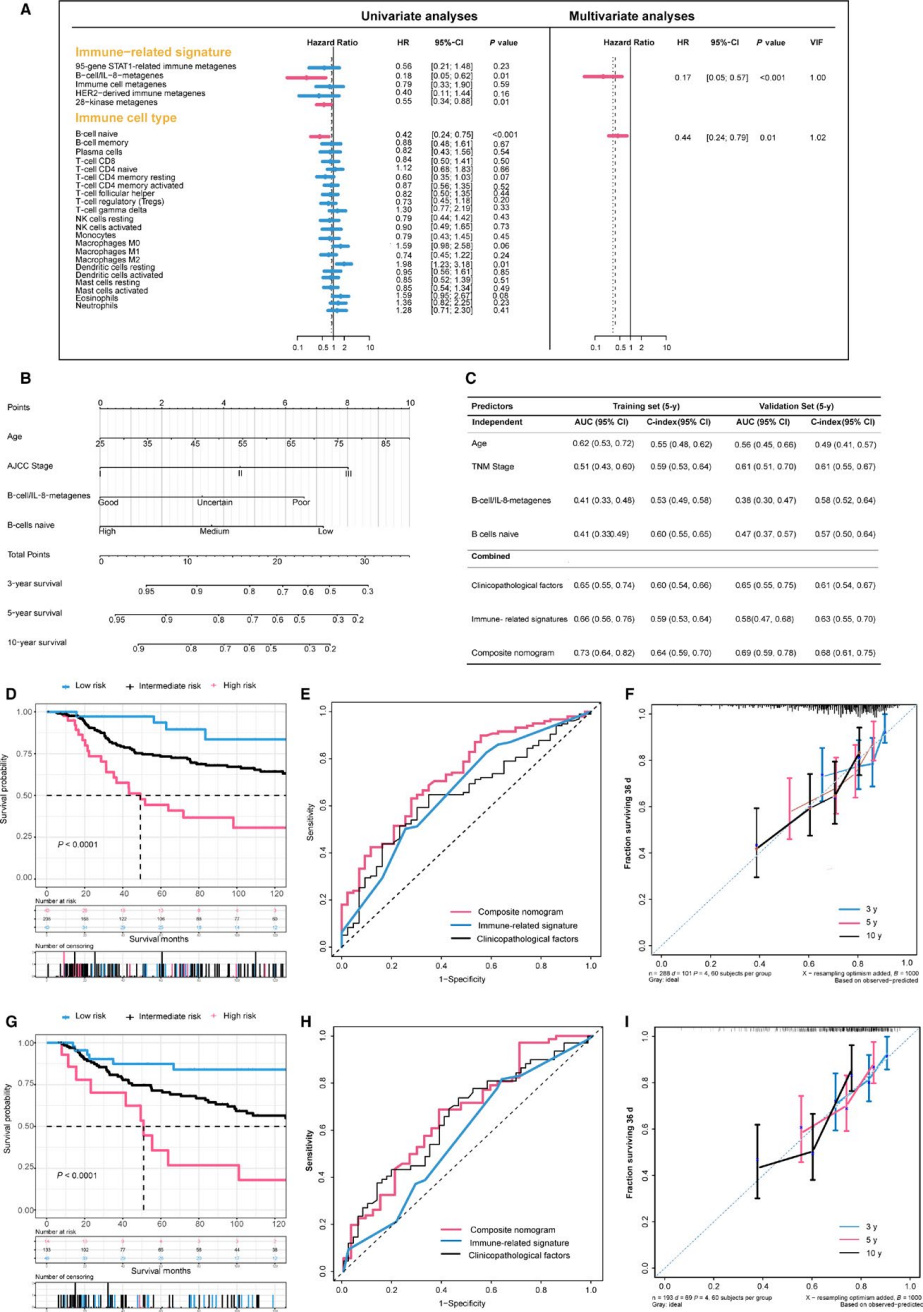
Development and validation of the composite clinicopathologic‐genomic nomogram for overall survival prediction. A, Analysis of prognostic performance using Cox regression; left: univariate analysis; right: multivariate backward stepwise Cox regression analyses in the training set. B, Composite nomogram to predict overall survival for TNBC. C, Performance of models and individual variables as assessed by 5‐year concordance index (C‐index) and area under the time‐dependent receiver operating characteristic (ROC) curve (AUC) in the training set and validation set for predicting overall survival for patients TNBC, whose 95% CIs were calculated from 1000 bootstraps of the survival data. D and G, Kaplan‐Meier survival curves of overall survival among risk stratification groups using the proposed nomogram in the training set (D) and the validation set (G). E and H, Time‐dependent ROC curves comparing the prognostic accuracies of 5‐year overall survival among the immune‐related genomic signatures combined with clinicopathologic features and the nomogram in the training set (E) and validation set (F). F and I, The calibration curves of the proposed nomogram for predicting overall survival (OS) at 3, 5, and 10 years in the training set (F) and in the validation set (I). Abbreviation: VIF, variance inflation factor

**Figure 3:**
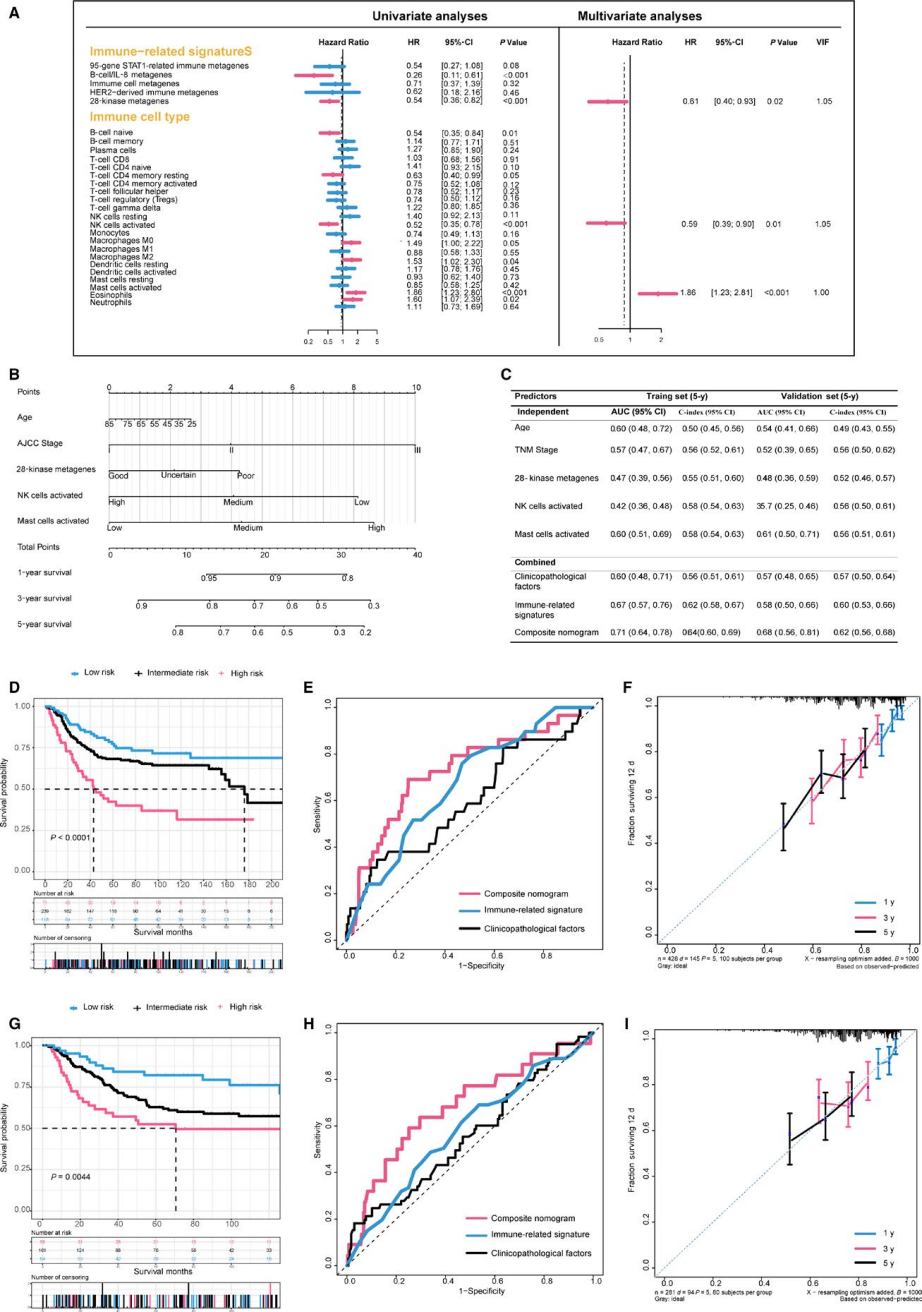
Development and validation of the composite clinicopathologic‐genomic nomogram for disease‐free survival prediction. A, Analysis of prognostic performance using Cox regression; left: univariate analysis; right: multivariate backward stepwise Cox regression analyses in the training set. B, Composite nomogram to predict disease‐free survival for TNBC. C, Performance of models and individual variables as assessed by 5‐year concordance index (C‐index) and area under the time‐dependent receiver operating characteristic (ROC) curve (AUC) in the training set and validation set for predicting disease‐free survival for patients TNBC, whose 95% CIs were calculated from 1000 bootstraps of the survival data. D and G, Kaplan‐Meier survival curves of disease‐free survival among risk stratification groups using the proposed nomogram in the training set (D) and the validation set (G). E and H, Time‐dependent ROC curves comparing the prognostic accuracies of 5‐year disease‐free survival among the immune‐related genomic signatures combined with clinicopathologic features and the nomogram in the training set (E) and validation set (F). F and I, The calibration curves of the proposed nomogram for predicting disease‐free survival (OS) at 1, 3, and 5 years in the training set (F) and in the validation set (I). VIF, variance inflation factor

Among 428 patients in the training set, 288 and 428 patients had survival data of OS and DFS, respectively. We sought to examine which factors were statistically significant for OS and DFS of patients with TNBC, respectively. Univariate Cox regressions of immune‐related signatures and OS or DFS were conducted firstly in training set, and we selected those statistically significant variables (*P* < 0.05) entering the stepwise multivariate Cox regression shown as Figures 2A and 3A.

We obtained different models integrating the immune‐related signatures identified before with clinicopathologic features according to OS and DFS, respectively. For endpoint of OS, older age (per year increase: hazard ratio (HR), 1.02; 95% confidence interval (CI), 1.00 to 1.03; *P* = 0.019), advanced stage (stage III vs I: HR, 2.72; 95% CI, 1.33 to 5.54, *P* = 0.006), poor prediction of B‐cell/IL‐8 metagenes (good vs. poor: HR, 0.19; 95% CI, 0.06 to 0.62, *P* = 0.006), and lower ratio of B cell naive (high vs low: HR, 0.40; 95% CI, 0.22 to 0.73, *P* = 0.003) were independently associated with inferior OS (Table S3). Likewise, the multivariate Cox regression for DFS indicated that TNM stage (stage III vs I: HR, 2.28; 95% CI, 1.34 to 3.88), prediction of 28‐kinase metagenes (good vs poor: HR, 0.64; 95% CI, 0.41 to 0.98, *P* = 0.041), proportion of activated NK cells (high vs low: HR, 0.55; 95% CI, 0.36 to 0.83, *P* = 0.005), and mast cells (high vs low: HR, 1.86; 95% CI, 1.22 to 2.81, *P* = 0.004) were independent prognostic variables (Table S3).

### 3.4 Construction, comparison, and validation of the composite nomogram

Above results were integrated and visualized as quantitative and user‐friendly nomograms (Figures 2B and 3B). Obviously, TNM stage and age at diagnosis had the largest contribution to DFS and OS, followed by immune‐related signatures and ratio of immune cells. Each category within these variables was assigned a point on the top scale based on the coefficients from multivariate Cox regression, and summed points of each patients as well as corresponding vertical line were obtained to predict the survival probability. The risk score cutoff values for OS (≤10.5, 10.5‐21, and ≥21) and DFS (≤11, 11‐22, and ≥22) were, respectively, selected on the basis of total points to divide patients into roughly equal tertiles in the training set, which accurately stratified patients into the low‐, intermediate‐, high‐risk subgroups (Figure 3). The patients in validation set were also divided into three subgroups in terms of the same cutoff values.

To assess additional value of immune‐related genomic information, we sought to compare the performance of the proposed nomograms with clinicopathological model and genomic model by applying time‐dependent receiver operating characteristic (ROC) analysis and C‐index to the training set and validation set. Expectedly, the composite nomogram had the greatest area under the ROC curve (AUC) and C‐index compared with clinical model and genomic model or single prognostic variables in both the training and validation sets (Figures 2 and 3). It is noteworthy that the nomogram for OS failed to be validated by validation set (AUC for nomogram, 0.69; AUC for clinical factors, 0.65; *P* = 0.96), due to limited sample size and overall deaths in validation set. The results indicated that the proposed nomograms had superior prognostic performance than either clinicopathological or immune‐related genomic information alone.

### 3.5 Associated biological pathways and immune checkpoint molecules

**Figure 4:**
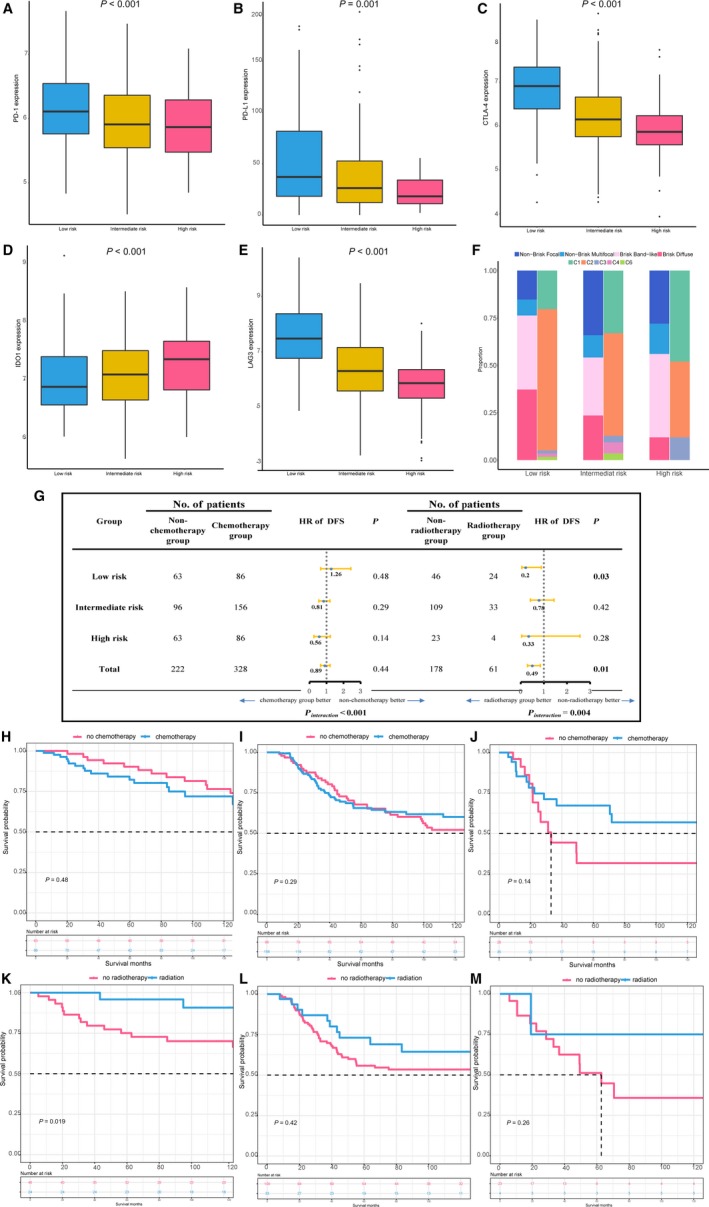
The nomogram discriminates survival benefit of chemotherapy and radiotherapy and ongoing immunotherapy. A‐E, Evaluation of the immune checkpoint expression (PD‐1, PD‐L1, CTLA‐4, IDO1, and LAG3) in different risk stratification groups defined by nomogram, where *P* values were derived from Mann‐Whitney *U* test. F, CNN‐derived tumor‐infiltrating lymphocytes (TILs) mapping identified from standard pathology cancer images by a deep‐learning‐derived ‘‘computational stain’’ developed by Saltz et al^67^ in different risk stratification groups defined by nomogram. Brisk diffuse: diffusely infiltrative TILs scattered throughout at least 30% of the area of the tumor; brisk, band‐like: immune responses forming band‐like boundaries bordering the tumor at its periphery; nonbrisk, multifocal: loosely scattered TILs present in less than 30% but more than 5% of the area of the tumor; nonbrisk, focal: TILs scattered throughout less than 5% but greater than 1% of the area of the tumor. Thorsson et al identified six immune subtypes (C1‐C6) to define immune response patterns impacting prognosis. C1, wound healing; C2, IFN‐g dominant; C3, inflammatory; C4, lymphocyte depleted; C5, immunologically quiet; C6, TGF‐b dominant. G, Hazard ratio comparing disease‐free survival between chemotherapy/radiotherapy group and nonchemotherapy/radiotherapy group according stratification groups defined by nomogram, respectively. H‐M, Kaplan‐Meier survival curves of disease‐free survival comparisons between chemotherapy/radiotherapy group and nonchemotherapy/radiotherapy group among risk stratification groups using the proposed nomogram

To reveal the potential biological meaning among final included immune‐related signatures, enrichment (GO) analyses were conducted. Figure S1 showed that specific GO categories closely related to TNBC prognosis, such as response to virus, nuclear nucleosome, regulation of lymphocyte activation, cytokine binding, focal adhesion, adaptive immune response (all FDR <0.0001), were significantly enriched. Additionally, we found that expression of several immune checkpoint molecules (ICMs), that is, programmed death‐1 (PD‐1), programmed death‐ligand 1 (PD‐L1), cytotoxic T‐lymphocyte‐associated protein 4 (CTLA4), an indoleamine 2,3‐dioxygenase 1 (IDO1), was significantly higher in the low‐risk group in whole cohort compared with intermediate‐ or high‐risk groups (Figure 4A‐E).

Additionally, we examined possible benefits from chemotherapy and radiotherapy in each subset defined by nomogram (Figure 4G‐M). Only low‐risk TNBC patients receiving radiotherapy were associated better DFS compared with those who did not receive radiotherapy (HR, 0.2, *P* = 0.03). Enslaved to sample size and missing chemotherapy data, this study just found decreased trend of nonsignificantly HRs comparing DFS between chemotherapy and non‐chemotherapy group from high‐risk (HR, 1.26, *P* = 0.48), intermediate‐risk (HR, 0.81, *P* = 0.29) to low‐risk group (HR, 0.56, *P* = 0.14).

## 4 DISCUSSION

There were challenges to predicting prognosis of patients with TNBC likely due to genetic heterogeneity both between and within tumors. Although an updated bioscore had been proposed within the context of the 8th edition American Joint Committee on Cancer (AJCC) staging system for breast cancer,^6^, ^51^ which substantially increased prognostic prediction of strength for breast cancer, TNBC patients could have remarkably different survival outcome even with the identical TNM stage.^44^ Herein, we integrated the large clinically annotated TNBC gene expression profiling datasets and separated it into training/validation sets to develop and validate a composite clinicopathologic immune‐related genomic nomogram for estimation of the risk of relapse/death in patients with TNBC. We found higher proportion of activated NK cells and naive B cell was associated with low risk of disease relapse and overall death in TNBC patients, respectively, whereas activated mast cells stood for worse prognostic indicators. The marginal trends were observed that low‐risk patients were likely to benefit from radiotherapy, whereas high‐risk individuals would have better survival due to adjuvant chemotherapy. Meanwhile, low‐risk subjects had higher expression of several ICMs, and immune checkpoint inhibitor might work well in this subgroup. This nomogram might also be used as a predictor of radiotherapy, chemotherapy, and upcoming popularity of immunotherapy.

Large amount of studies had revealed the association between TILs and cancer progression and patient survival, such melanoma and ovarian, breast, bladder, cervical, colon, prostate, rectum, and lung cancers.^52^, ^53^, ^54^, ^55^, ^56^ It was well known that TILs enclose cytotoxic CD8+ T cells, CD4+ T‐helper cells (Th), CD4+/FOXP3+ regulatory T cells (Treg), B cells and NK cell, and T lymphocytes are the most predominant type of lymphocytes in the microenvironment.^57^ Rich CD8+ T lymphocytes in TNBC were associated with a better prognosis,^58^ whereas the similar phenomenon could not be observed for ER‐positive breast cancer.^59^ CD4+ T lymphocytes are stratified into T‐helper cells (TH1), Tfh, and regulator T lymphocytes. Both TH1 and Tfh were associated with better prognosis in ER‐positive cancer, but not in TNBC.^57^ Iglesia et al showed that B‐cell gene expression signature was associated with better DFS for basal‐like and HER‐2 enriched cancers,^60^ which was comparable with our results that B cell naive was a good indicator for DFS. Similarly, previous studies showed that breast tumor progression involves natural killer cells dysfunction and that breast tumors model their environment to evade NK cell antitumor immunity.^61^, ^62^ Studies of breast cancer have shown natural killer cells to be associated with a better prognosis, still little is known about interaction effect between natural killer cells and breast cancer subtypes.^63^, ^64^ Interestingly, another research^65^ also used CIBERSORT to estimate the fraction of 22 immune cell types to study their relations with pCR, DFS, and OS of breast cancer patients finds that in the TNBC subtype, a higher fraction of resting NK cells was associated with worse DFS and OS, and a higher fraction of plasma cells was associated with improved DFS. Although this study had larger sample size than us, they employed the multivariable Cox regression to assess the associations of fraction of 22 immune cell types and survival outcomes at one time, neglecting the potential effects of collinearity. In the contrast, we conducted univariable Cox regression to select the significantly prognostic immune cells, which were further analyzed using multivariate backward stepwise Cox regression, where effects of collinearity were measured with variance inflation factor.

Recent studies further documented that the spatial context and the nature of cellular heterogeneity of the tumor microenvironment also had effect on prognosis.^39^, ^66^, ^67^ Different mechanisms between the intra‐ and peri‐tumoral processes in the immune system had been identified. The intra‐tumoral process often referred the concept of “immunoediting,” which can well present the tumor‐sculpting and host‐protecting action of the immune system, including three phases: elimination, equilibrium, and escape.^68^ The representative example in peri‐tumoral process is TILs, high densities of which correlated with favorable survival outcomes in multiple cancer types.^69^ When we stratified patients with TNBC from TCGA into four “spatial subtype” (brisk diffuse; band‐like; nonbrisk, multifocal; and nonbrisk, focal) according to TIL maps constructed before^67^ (Figure 4F), intermediate‐ and high‐risk individuals, who tended to have poor prognoses, had greater proportion of “nonbrisk” types, which was consistent with expectations that the relatively low degree of lymphocytic infiltrates predicted poor clinical outcomes. Interestingly, mirrored results were also seen in distribution of immune subtypes defined in the TCGA pan‐immune analysis,^66^ C1 and C2 having a substantial immune component are main immune phenotypes in TNBC (Figure 4F), which were associated with improved outcomes when higher lymphocyte signatures existed.^66^ Additionally, the C4 subtype that is relatively richer in cells of the monocyte/macrophage lineage is consistently remained in the low‐ and intermediate‐risk group rather than high‐risk individuals.

In light of TNBC‐specific immunogenic and actively engagement by the immune system, immunotherapies are testing among a population of TNBC. A phase I trial (KEYNOTE‐012) enrolled patients with advanced stage TNBC and positive PD‐L1 expression, and a response rate of 18.5% was observed after treated with the anti‐PD‐1 monoclonal antibody pembrolizumab.^70^ In another ongoing phase III trial NeoTRIPaPDL1 (NCT02620280), patients with locally advanced TNBC will be randomly assigned to receive nab‐paclitaxel and carboplatin with or without a PD‐L1‐inhibitor (atezolizumab), and the endpoint of this trial was event‐free survival. Pending confirmation through randomized, controlled clinical trials in other cancers,^71^, ^72^, ^73^ some of these studies indicated that chemotherapy and immunotherapy can create a synergy. Indeed, we found TNBC patients with high ICMs expression in our low‐risk subgroup would not benefit from chemotherapy and immunotherapy at the same time, suggesting mechanisms of this phenomenon should be further studied in TNBC. Interestingly, although those related trials enrolled patients into experimental group based on PD‐L1 expression, some solid malignancies patients with PD‐L1‐negative received PD‐1‐targeting antibody nivolumab also yielded substantial survival advantages.^74^ Thus, next‐generation immunotherapy beyond checkpoints was establishing, and various combinations of checkpoint targeting agents are also being investigated in preclinical and clinical trials.^75^, ^76^, ^77^, ^78^ Additionally, radiotherapy can increase the production of cytokines and activate antitumor immune response, inhibiting the proliferation of tumor cells.^79^, ^80^ A wealth of pre‐clinical data demonstrates that radiotherapy potentiates the activity of a diverse range of immunotherapies.^81^ In our study, we found that low‐risk TNBC patients defined by nomogram were associated with higher ICMs expression and had better survival after radiotherapy, indicating immune checkpoint inhibitor combined with radiotherapy might work well in this subgroup.

Although it was reported that previous immune‐related genomic signatures associated with survival prognosis in their respective publications (Table S2), only B‐cell/IL‐8 metagenes^20^ and 28‐kinase metagenes^21^ were included in the final model. This may partially explain wide nonoverlapping among the immune‐related signatures identified in TNBC microarray studies, and no association between them was found. This is noteworthy, as these signatures might reflect different biological behavior, and varied result was also due to interpatient heterogeneity. Of note, 95‐gene STAT1‐related immune signature was enriched for genes reflecting response and defense response to virus.^17^ Accordingly, during procedure of chronic virus infections (eg, HIV, hepatitis B), T cells progressively lose responsiveness, amounts of T cells are eliminated by apoptosis, and the living T cells are limited in a functionally impaired, causing the inhibition of antitumor immunity.^82^ Moreover, a recent clinical trial with anti‐PD‐1, PD‐L1, and CTLA‐4 showed the concept that immunity to chronic virus can be improved by interfering with inhibitory pathways.^83^ Likewise, the association between HER2‐derived signature^22^ and cytokine binding was also found. Over the past decades, using the possibility of recombinant cytokines as a trigger or boost in anticancer immunity has been interested.^84^, ^85^, ^86^ Nevertheless, only three agents have been proved: recombinant interleukin (IL)‐2 and two variants of recombinant alpha 2 (IFN‐α2), namely IFN‐α2a and IFN‐α2b,^87^ and they exhibit a relatively restricted cell specificity,^88^ immune checkpoint blocker might induce these cytokine binding to magnify the anticancer effect. In the view of these, immune‐related genomic signatures for TNBC should be constructed in a comprehensive approach in the future.

Despite the promising results, several limitations should be addressed in this study. First, the NTP method uses only a list of signature genes to make class predictions in each patient's expression data, which allowed the method to be less sensitive to differences in experimental and analytical conditions and applicable to every patient without optimization of the analysis parameters.^39^ Also, we evaluated several immune cells from CIBERSORT instead of evaluating them all, some important immune phenotypes like myeloid‐derived suppressor cells would be missed.^89^ As the average follow‐up was 30 months for TNBC patients, another limitation is the relatively short average follow‐up for each patient. Of note, since the OS data were unavailable in 30% cases, it was not reliable for OS analysis. Indeed, patients with ER‐negative disease often experience relapse within the first 5 years,^90^ and limited disease‐related events lead to inadequate comparison for each variable. Finally, the nomograms reported here are little complex, and better screening method could be used to integrate those important immune signatures and fulfill the immunotype classification.

In conclusion, this is the first study evaluating and integrating the immune‐related genomic signatures of TNBC with clinicopathologic features, and the improved performance of this combinatorial scheme accentuated the importance of integrating all aspects of the immunogenic and immune microenvironmental features into prognostic stratification. Moreover, this nomogram could be a useful prognostic and predictive tool to identify patients with TNBC who might benefit from adjuvant chemotherapy, radiotherapy, and upcoming immunotherapy, which might have crucial implications for the postoperative personalized follow‐up and decision‐making regarding individualized adjuvant treatment.

## CONFLICT OF INTERESTS

The authors declare no potential conflict of interests.

## COMPLIANCE WITH ETHICAL STANDARDS

All of those samples previously were approved by their respective institutional reviews boards.
