# Supplementary figures and images for: Development and validation of nomograms integrating immune‐related genomic signatures with clinicopathologic features to improve prognosis and predictive value of triple‐negative breast cancer: A gene expression‐based retrospective study
Source: Cancer Med. 2019 Jan 24;8(2):686–700. doi: 10.1002/cam4.1880 (PMC6382728; doi:10.1002/cam4.1880)

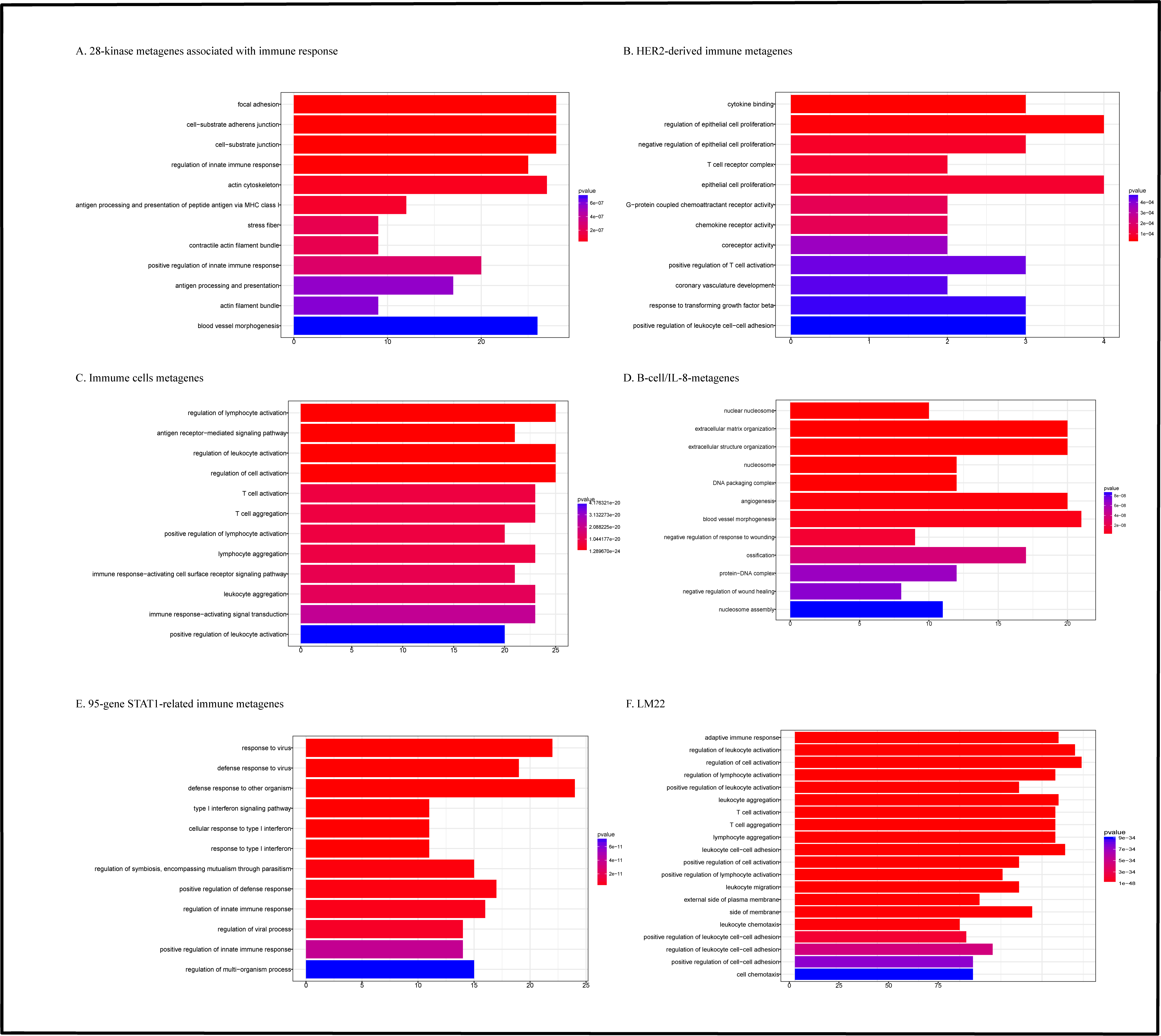

Supplement: Supplementary file 1 [file CAM4-8-686-s001.tif]

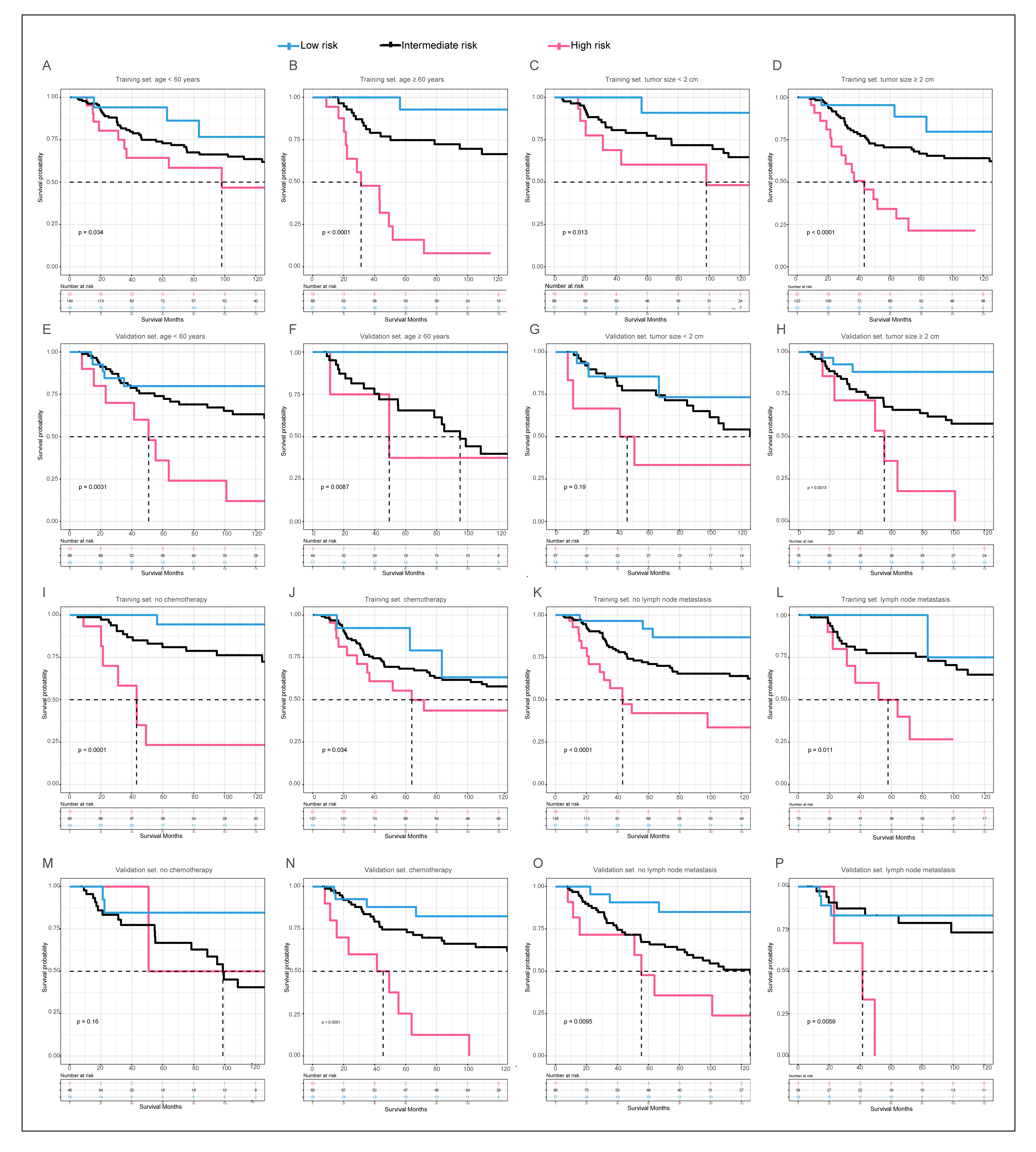

Supplement: Supplementary file 2 [file CAM4-8-686-s002.tif]

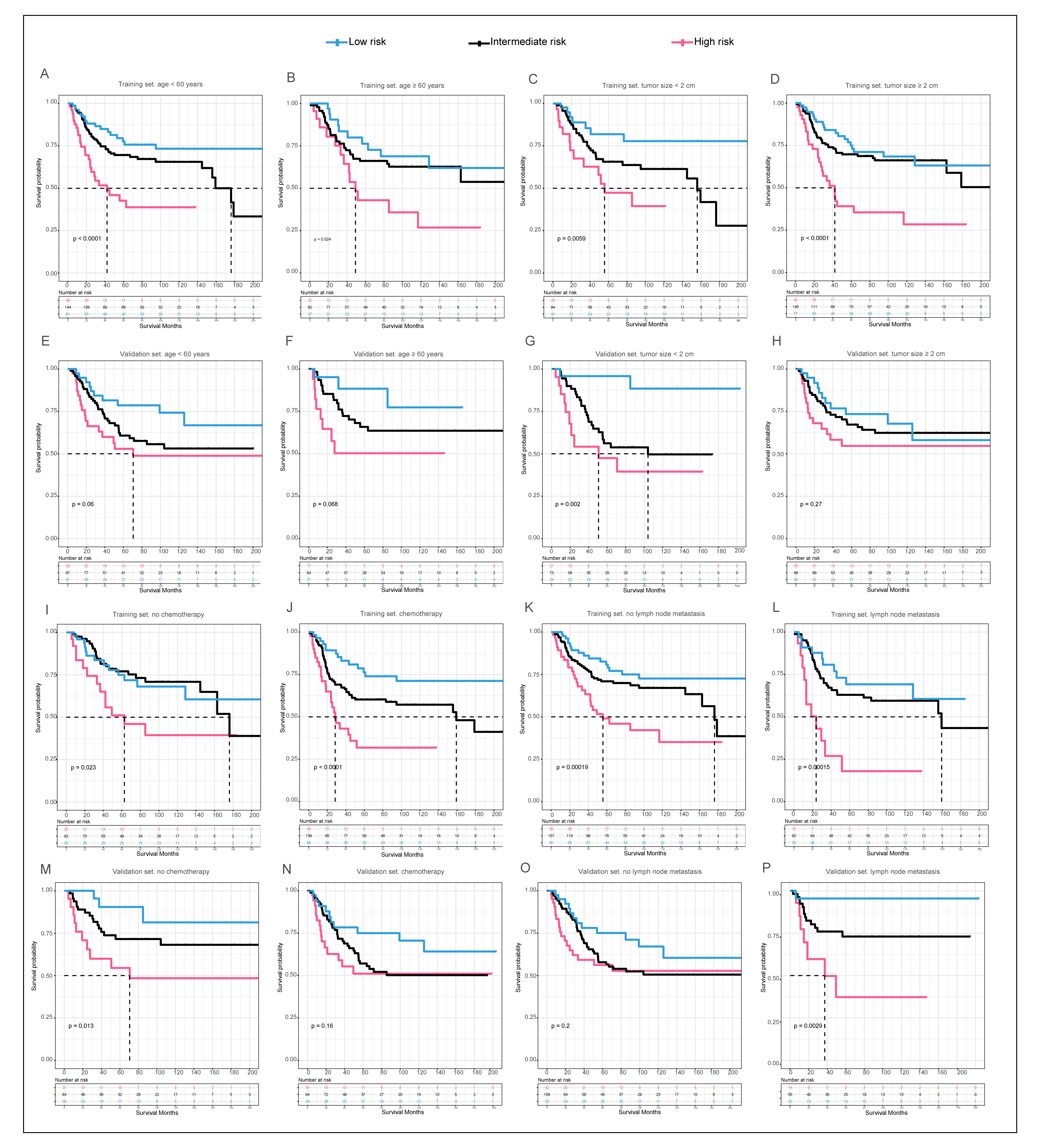

Supplement: Supplementary file 3 [file CAM4-8-686-s003.tif]
