# Supplementary material for: Development and validation of nomograms integrating immune‐related genomic signatures with clinicopathologic features to improve prognosis and predictive value of triple‐negative breast cancer: A gene expression‐based retrospective study
Source: Cancer Med. 2019 Jan 24;8(2):686–700. doi: 10.1002/cam4.1880 (PMC6382728; doi:10.1002/cam4.1880)
Supplement: Supplementary file 4 [file CAM4-8-686-s004.docx]

**Supplementary TableS1 Summary of included studies.**

| **Study name** | **Accession number/Source** | **PMID** | **Platform** | **Number of TNBC/BC (%)** | **False positive (%)** | | |
| --- | --- | --- | --- | --- | --- | --- | --- |
|  |  |  |  |  | **ER** | **PR** | **HER2** |
| DFHCC | GSE19615 | 20098429 | Affymetrix HGU | 30/115(26) | 0 | 0 | 6.1 |
| EMC2 | GSE12276 | 19421193 | Affymetrix HGU | 42/204(21) | NA | NA | NA |
| MAINZ | GSE11121 | 18593943 | Affymetrix HGU | 13/200(21) | 1 | NA | NA |
| PNC | GSE20711 | 21910250 | Affymetrix HGU | 18/92(20) | 2.2 | 8.7 | 2.2 |
| STK | GSE1456 | 16280042 | Affymetrix HGU | 23/159(14) | 4.4 | NA | NA |
| TRANSBIG | GSE7390 | 17545524 | Affymetrix HGU | 25/198(13) | 1 | NA | NA |
| UNT | GSE2990 | 16478745 | Affymetrix HGU | 20/92(22) | 3.3 | 5.4 | NA |
| UPP | GSE3494 | 16141321 | Affymetrix HGU | 23/190(12) | 6.3 | 5.3 | NA |
| VDX | GSE2034/GSE5327 | 17420468 | Affymetrix HGU | 79/344(23) | 2.3 | NA | NA |
| TCGA | TCGA | 26451490 | RNA-seq | 198/1095(18) | 8.4 | 12.6 | 6.3 |
| METBRC | METBRC | NA | RNA-seq | 240/1904(13) | 0.2 | 0 | 0.1 |

Abbreviation: TNBC, triple negative breast cancer; BC, breast cancer; NA, not available.

**Supplementary FigureS1** GO analyses of 6 immune-related genomic signatures included in this study.

**Supplementary FigureS2** Kaplan-Meier survival curves of overall survival for patients in the training set and validation set.

**Supplementary FigureS3** Kaplan-Meier survival curves of disease-free survival for patients in the training set and validation set.
