# Supplementary material for: Development and validation of nomograms integrating immune‐related genomic signatures with clinicopathologic features to improve prognosis and predictive value of triple‐negative breast cancer: A gene expression‐based retrospective study
Source: Cancer Med. 2019 Jan 24;8(2):686–700. doi: 10.1002/cam4.1880 (PMC6382728; doi:10.1002/cam4.1880)
Supplement: Supplementary file 5 [file CAM4-8-686-s005.docx]

**Supplementary TableS2 Initial Immune-related Genomic Signatures in the Study**

| **N0** | **Signature name** | **Author/year** | **TNBC definition** | **No. of genes** | **Immune-related gene signature** | |
| --- | --- | --- | --- | --- | --- | --- |
|  |  |  |  |  | **Favorable prognosis** | **Poor prognosis** |
| 1 | T-cell-related metagene | Callari et al. (2016) [1] | ER-negative/ HER2-negative | 6 | CXCL13, PRF1, IRF1, IKZF1, GZMB, HLA-E | |
| 2 | STAT1-related immune metagene | Desmedt et al. (2008) [2] | ER-negative/ HER2-negative | 95 | STAT1, CXCL10, TAP1, CXCL11, INDO, CXCL9, MX1, LAMP3, ISG15, RTP4, HERC6, IFI44L, MX2, IFIT3, HERC5, RSAD2, DDX58, CCL5, ADAMDEC1, CD2, NA, HCP5, NMI, SPOCK2, CCL8, TRIM22, LYZ, IRF1, LAG3, PSCDBP, TFEC, UBD, SP140, CTSC, IFI6, PLA2G7, CD3G, ECGF1, PLAC8, FGL2, GZMK, CD48, STAT4, GPR18, P2RX5, IFI30, SH2D1A, LAPTM5, CD69, PTPN7, IRF8, PIM2, ETV7, GPR171, PSME1, BIRC3, FASLG, IFITM1, IFIT5, ITGB2, BTN3A2, HCLS1, SECTM1, ARHGAP15, KLRK1, IGSF6, EBI2, NA, SNX10, NA, BST2, NA, APOC1, NA, NA, ZC3HAV1, LILRA4, EBI3, KLRC3, CLEC4A, CD40LG, VAV1, GLRX, ACP5, RFX5, CECR1, TRAF3, RAB8A, IL18, RASGRP1, REC8L1, CCRL2 | DDAH2, EFNA1, DNAL4 |
| 3 | Follicular helper T (TFH)-cell signature | Gu‑Tranti-en *et al.* (2013)[3] | ER‑negative/ HER2‑negative | 8 | CD200, CXCL13, FBLN7, ICOS, SGPP2, SH2D1A, TIGIT, PDCD1 | |
| 4 | Several immune-related genes | Denkert et al.  (2015)[4] | ER‑negative/ HER2‑negative | 12 | CCL5, CXCL9, CXCL13, CD8A, PD1, PDL1, CTLA4, FOXP3, IDO1, IGKC, CD80, CD21 | |
| 5 | Prognostic immune-related module | Teschendo-rff *et al.* (2007) [5] | ER‑negative/ HER2‑negative | 7 | C1QA, IGLC2, LY9, TNFRSF17, XCL2, HLA-F, SPP1 | |
| 6 | HER2‑derived prognostic predictor-enriched in immune genes | Staaf *et al.* (2010) [6] | Basal-like breast cancer | 14 | STAT5A, TGFBR3, SOCS1, GPC3, CX3CR1, SPRY2, RUNX3, CD69, CD3D, CD247 | CXCR4, PLAU, CD55, SPP1 |
| 7 | B-cell/IL-8 metagenes | Rody *et al.* (2011) [7] | ER‑negative/ HER2‑negative | 244 | GPR20, IGH@, XCL1, TRA@, KLRD1, IGL@, CXCL9, AMY2A, ZNF3, IGLC2, BMP7, FAS, LOC391427, CDC42EP4, LPIN1, ALDH5A1, DOK4 | IL8, SCD, AQP3, IL8, SERPINE1, LYPDC1, PGK1, SEC23A, WASL, PGK1, HIST1H2BG, SULF1, SPP1, TREM1, GCNT3, IDI1, ZCCHC14, MAGEA2, PNAS-4, TPM2, GREM1, IDI1, SULF1, GREM1, SULF1, SLC2A3, HMGCR, HIST1H3D, KIAA1199, NOX4, MMP10, ADM, THBS1, HIST1H2BG, IL1RAP, NP, APOA1, THBS1, LOX, SNAPC1, LPA, TGFBI, HIST1H3C, C14orf111, DKFZP586J0619, WWTR1, SCD, FLJ10407, SEC23A, SLCO1B3, EMP1, SEC24A, PPFIA4, RGS4, LAMA4, KRT6A, AQP3, RPESP, RNF141, PDE4D, PGK1, CDYL, ZP2, ACY1, |
|  |  |  |  |  |  | ADAM10, ULBP2, H2BFL, ALPPL2, PLAUR, ACTN1, HIST1H2BB, CYTL1, PFKP, HIF1A, HIST1H2BF, LOX, IL13RA1, CSNK1G3, TNFRSF10B, CRLF1, BAG3, LRRIQ2, SERPINE1, GADD45B, TRIM23, TIAM1, EMP1, AVEN, NBR2, CA9, PHLDA2, SRPX2, KIAA0217, SYNCRIP, HUS1, HIST1H2BE, RUNX1, MAGEA6, VMD2, U2AF1L1, COL10A1, ELAVL2, F2RL1, H4FH, CUL4B, STMN2, RGS2, HAPLN1, LOC201501, HES1, FN1, ACOX1, MMP1, TSGA10, LAMA4, CUL4B, FAM12B, AIM1, SARG, FUT9, ZA20D1, THBS1, ESM1, MVK, PDK1, PPEF1, ABHD6, RGS4, IL1RAP, BHLHB2, IFNA2, LOC442165, UQCRC1, |
|  |  |  |  |  |  | HIST1H2BI, LMX1B, IBSP, RAB5A, CTAG2, RUNX1, RAB3B, MYT1L, IL24, ALDOAP2, TLE6, ALOX15, SPOCK, GMFB, GCP2, JAG1, NAT6, CTGF, TXNDC9, CLIC4, LOX, CBFB, EGLN3, DDX31, HMGCR, HIST1H1C, NMD3, DLGAP1, PTPN21, FDPS, SERPINB2, ANP32A, CBX3, RODH, GPCR5A, FLJ21934, CSPG2, SCC-112, KPNA1, FLNB, FLJ11004, TNFAIP1, VLDLR, TGFB2, MAGEA12, BUCS1, ADFP, PSG1, ANXA2, HIST1H2BH, SLC27A5, DSCR1, CDC2L5, PLAU, AEBP1, NNMT, SNF7DC2, IL13RA1, ABHD6, ANGPTL4, DDX51, AMD1, ENPEP, HIST1H3G, TAX1BP3, SORT1, STC1, LOC51035, PTPN12, PP1345, HIST1H2AE, PLAU, C14orf92, CYP51A1, |
|  |  |  |  |  |  | HYAL1, HIST1H2BO, TBC1D22B, WFDC1, MCCC2, C19orf6, KCNE4, PLAUR, NLGN4X, CALML5, DBC1, CTAG1B, SLC25A30, NPC1L1, KIAA0406, CUL4B, NF1, RBMS2, ITGB6, CLDN1 |
| 8 | 28‑kinase metagene associated with immune  response | Sabatier et al. (2011) [8] | Basal-like breast cancer | 368 | GTSE1, NCF1, ITGB7, FBXL14, PPP1R15B, TRAF3, C11orf31, C1S, NOC3L, MAP2K1, PNKD, IFI30, HERPUD1, LOC642031, TAGAP, CCDC69, SEC23IP, SGPL1, SLC4A1AP, CASP10, MYNN, SLC7A1, PSMB10, NUP93, CHCHD1, BUB3, CYBA, SRGN, EPB41, ADORA2A, DAZAP1, ICAM1, NSMCE4A, ZNF512B, ELL, SOCS3, RILPL2, PSAP, NOP16, RNF207, RARS, SNHG12, HSPA4, ERO1LB, DHX34, MINPP1, SIAH2, ZMIZ2, C12orf4, CLEC2D, IL32, SCML1, MICB, ANXA7, RASGEF1A, IL12RB1, ZFP42, ABCA3, TRAFD1, C1orf174, PQBP1, CBS, KTELC1, BTN3A3, FOXJ2, CCDC88C, UNQ2963, SLC2A6, TIAL1, ATP8A1, FBL, PARP8, M6PR, IGL@, NFE2L3, LAG3, SOD2, NME3, C2CD2, AIM2, PSMG4, PRR13, DTX3L, PIM2, PUS1, XPNPEP1, C9orf46, PARVB, RPS21, BTN3A1, ISCU, SKAP1, UBD, FOXM1, NFKBIE, EMG1, PHB2, RIMKLB, TYSND1, PSMB9, NBN, TAP1, TNFRSF1B, APOE, PSMB2, FAM53B, IL15, PHF19, CD38, C12orf11, ADAM8, GABPB2, ADRBK2, TLR2, CEBPD, BIRC3, KLRC1, RELB, KBTBD8, STAT1, GALM, PPIF, GBP4, LAP3, TREX1, CTSS, GZMA, PCOLCE2, CSDA, PCSK6, PPA1, CEP55, DDX50, VCAM1, MKI67, OVOS2, TNFRSF14, ABCC4, ZCCHC9, WARS, MRTO4, ECE2, HLA-DOB, ETV7, HSH2D, RGS1, HEATR1, LARS, EME2, ARNTL2, PSMA5, PSMB8, CDCA3, CDCP1, CASS4, MIRN155, TYMP, STAMBPL1, PLCL2, NOP2, GLRX, PTPN6, | \| SMTN, BOC , ACTG2, MPRIP, KANK2, KIDINS220, MYLK, SH3PXD2A, MYL9, DAAM1, TAGLN, GSTA1, PTRF, NCOA1, ACTN1 , C1orf116, TTC28, TPM2, OBSL1, LOC654342, VSNL1, EFNA3, FSTL1, CTNND2, LOC728264, FHL1, HIST1H4H, TMEM134, PCDH18, PARVA, SLC4A3, C20orf108, SORT1, EXTL2, ACTA2, CALD1, HIST1H2AC, UACA, SPEG, CAPN2, FERMT2, RTN4, DDR2 , ZNF385D, FKBP9, ANO1, MXRA7, NCRNA00086, H19, SNAI2, COPZ2, ZCCHC24, IPW, HIST1H4J, TMEM61, TGFB1I1, AQP1 , TIMP3, CADM1, KCNMB1, LOH3CR2A, TUBG2, MXRA8, EDN2, CAPN5, SPOCK1, PICALM, DACT3, FLNA, FAM101B, TSGA14, GTF2I, HSPC159, NPR2, EDNRA, KIAA1324L, STARD13, SNRPN, PERP, \| \| --- \| |
|  |  |  |  |  | C10orf119, FAM26F, STYK1, IL15RA, ACSL5, TNFAIP2, E2F5, RAP2B, IQCG, RCL1, DNAJC9, TEAD4, CENPA, MCM10, TAPBPL, CUL2, SPATA5L1, IRF1, RFX5, CLEC7A, AIFM2, TRABD, NR1H3, DRAM, ANKRD22, GYG1, TCOF1, ASPHD2, ME2, MAGOHB, C9orf72, GBP5, FAM91A2, ERAP1, GRWD1, IL27RA, NFKB2, C6orf150, C1RL, ETV6 | COPS8, LOC645166, PRICKLE1, THBS4, RGS5, HIST1H2AE, BGN, MTSS1, ITGB5, RABGAP1, RUNX1, C20orf194, TTC3, FBXW2, PPP1R1B, PTPN21, ACVR1, PLSCR3, MYH10, CD81, NAV3, LOC647979, CACHD1, RIT1, ATL1, MAPK7, HSPG2, C11orf80, VPS24, MBOAT2, LIMS2, VPS37D, MSRB3, SLIT3, RCAN1, EPHA3, PDGFD, ERBB4, SRPX2, TLN2, CAV1 , KLHDC10, AGPAT2, PALM2, LIN28, DLC1, SEMA5A, PTPRZ1, MAGI3, CCDC15, CNIH3, MYH9, KCTD1, CX3CR1, FAP, SPRED2, FAM69B, ENPP1, LGR6, NOSTRIN, ZNF20, AHNAK, KATNAL1, C9orf89, ADAMTS12, TMEM192, ACSL3, NDUFV1, DDAH1, LAMA2 , ZFYVE1, PDE5A, |
|  |  |  |  |  |  | CNPY4, SAV1, MRVI1, RGS4, CNN2, FLNC, ENDOD1, AOC3, C9orf3, MDFI, NPR3, MMGT1, GPC1, NME7, SNX21 |
| 9 | Immune cells metagenes | Nagalla *et al.* (2013)  [9] | basal-like breast cancer | 70 | HLA-E, HLA-DPB1, SRGN, TNFAIP3, HCLS1, CSF1R, INPP5D, CD4, PIK3CD, CYBB, HLA-DMB, CD48, MFNG, ALOX5AP, CD37, GMFG, IRF4, SELL, HLA-DRB1, EVI2A, FGL2, ARHGAP25, LCK, IL10RA, SASH3, PTPRCAP, CD79A, CSF2RB, POU2AF1, CD3E, GZMA, CD27, CCR7, TNFRSF17, GZMK, CCR2, PTPRC, LTB, LAX1, PRKCB, HLA-DRA, CORO1A, CKAP2, IGHG1, CD74, TRAC, YME1L1, IL16, CD247, SH2D1A, GPR18, LST1, TRBC1, ITK, IGHV1-69, IGHA1, IGKC, IGKV3-20, EVI2B, FYB, IL23A, HLA-DPA1, IGJ, HLA-DQA1, HLA-DQB1, DOCK2, LOC91316, CD3D, RAC2, NKG7, CTSW, KLRB1, XCL2, FAM20B, IGKV4-1, IGHD, NTN3, IGLJ3, IGLL3, IGKV1D-13, LPXN, LOC652493, LOC100290557, IGLV1-44, IGHM, IGL@, LOC100130100, IGHV3-23, HLA-DMA, LOC100287723, GIMAP5, ARHGAP15, GIMAP6, PVRIG, LAT2 | CKAP2, FAM20B, YME1L1 |
|  |  |  |  |  |  |  |

[1] M. Callari, V. Cappelletti, F. D'Aiuto, V. Musella, A. Lembo, F. Petel, T. Karn, T. Iwamoto, P. Provero, M.G. Daidone, L. Gianni, G. Bianchini, Subtype-Specific Metagene-Based Prediction of Outcome after Neoadjuvant and Adjuvant Treatment in Breast Cancer, Clin Cancer Res 22(2) (2016) 337-45.

[2] C. Desmedt, B. Haibe-Kains, P. Wirapati, M. Buyse, D. Larsimont, G. Bontempi, M. Delorenzi, M. Piccart, C. Sotiriou, Biological processes associated with breast cancer clinical outcome depend on the molecular subtypes, Clin Cancer Res 14(16) (2008) 5158-65.

[3] C. Gu-Trantien, S. Loi, S. Garaud, C. Equeter, M. Libin, A. de Wind, M. Ravoet, H. Le Buanec, C. Sibille, G. Manfouo-Foutsop, I. Veys, B. Haibe-Kains, S.K. Singhal, S. Michiels, F. Rothe, R. Salgado, H. Duvillier, M. Ignatiadis, C. Desmedt, D. Bron, D. Larsimont, M. Piccart, C. Sotiriou, K. Willard-Gallo, CD4(+) follicular helper T cell infiltration predicts breast cancer survival, J Clin Invest 123(7) (2013) 2873-92.

[4] C. Denkert, G. von Minckwitz, J.C. Brase, B.V. Sinn, S. Gade, R. Kronenwett, B.M. Pfitzner, C. Salat, S. Loi, W.D. Schmitt, C. Schem, K. Fisch, S. Darb-Esfahani, K. Mehta, C. Sotiriou, S. Wienert, P. Klare, F. Andre, F. Klauschen, J.U. Blohmer, K. Krappmann, M. Schmidt, H. Tesch, S. Kummel, P. Sinn, C. Jackisch, M. Dietel, T. Reimer, M. Untch, S. Loibl, Tumor-infiltrating lymphocytes and response to neoadjuvant chemotherapy with or without carboplatin in human epidermal growth factor receptor 2-positive and triple-negative primary breast cancers, J Clin Oncol 33(9) (2015) 983-91.

[5] A.E. Teschendorff, A. Miremadi, S.E. Pinder, I.O. Ellis, C. Caldas, An immune response gene expression module identifies a good prognosis subtype in estrogen receptor negative breast cancer, Genome biology 8(8) (2007) R157.

[6] J. Staaf, M. Ringner, J. Vallon-Christersson, G. Jonsson, P.O. Bendahl, K. Holm, A. Arason, H. Gunnarsson, C. Hegardt, B.A. Agnarsson, L. Luts, D. Grabau, M. Ferno, P.O. Malmstrom, O.T. Johannsson, N. Loman, R.B. Barkardottir, A. Borg, Identification of subtypes in human epidermal growth factor receptor 2--positive breast cancer reveals a gene signature prognostic of outcome, J Clin Oncol 28(11) (2010) 1813-20.

[7] A. Rody, T. Karn, C. Liedtke, L. Pusztai, E. Ruckhaeberle, L. Hanker, R. Gaetje, C. Solbach, A. Ahr, D. Metzler, M. Schmidt, V. Muller, U. Holtrich, M. Kaufmann, A clinically relevant gene signature in triple negative and basal-like breast cancer, Breast cancer research : BCR 13(5) (2011) R97.

[8] R. Sabatier, P. Finetti, N. Cervera, E. Lambaudie, B. Esterni, E. Mamessier, A. Tallet, C. Chabannon, J.M. Extra, J. Jacquemier, P. Viens, D. Birnbaum, F. Bertucci, A gene expression signature identifies two prognostic subgroups of basal breast cancer, Breast cancer research and treatment 126(2) (2011) 407-20.

[9] S. Nagalla, J.W. Chou, M.C. Willingham, J. Ruiz, J.P. Vaughn, P. Dubey, T.L. Lash, S.J. Hamilton-Dutoit, J. Bergh, C. Sotiriou, M.A. Black, L.D. Miller, Interactions between immunity, proliferation and molecular subtype in breast cancer prognosis, Genome biology 14(4) (2013) R34.
