# Supplementary material for: Development and validation of nomograms integrating immune‐related genomic signatures with clinicopathologic features to improve prognosis and predictive value of triple‐negative breast cancer: A gene expression‐based retrospective study
Source: Cancer Med. 2019 Jan 24;8(2):686–700. doi: 10.1002/cam4.1880 (PMC6382728; doi:10.1002/cam4.1880)
Supplement: Supplementary file 6 [file CAM4-8-686-s006.docx]

| **Table S3 Multivariable Cox regression for patients with TNBC in training set.** | | | | |
| --- | --- | --- | --- | --- |
| **Variable** | Multivariate Analysis (OS) | | Multivariate Analysis (DFS) | |
|  | HR (95% CI) | P value | HR (95% CI) | P value |
| **Age at diagnosis (years) †** | 1.02 (1.00, 1.03) | **0.019** | 1 (0.98, 1.01) | 0.563 |
| **AJCC Stage** |  |  |  |  |
| I | Reference |  | Reference |  |
| II | 1.76 (1.08 , 2.87) | **0.024** | 1.43 (0.98 , 2.09) | 0.061 |
| III | 2.72 (1.33, 5.54) | **0.006** | 2.28 (1.34 , 3.88) | **0.002** |
| **B-cell/IL-8 metagenes** |  |  |  |  |
| Poor | Reference |  |  |  |
| Uncertain | 0.99 (0.63, 1.54) | 0.952 |  |  |
| Good | 0.19 (0.06, 0.62) | **0.006** |  |  |
| **28-kinase metagenes** |  |  |  |  |
| Poor |  |  | Reference |  |
| Uncertain |  |  | 1.16 (0.79, 1.71) | 0.441 |
| Good |  |  | 0.64 (0.41, 0.98) | **0.041** |
| **B cells naive** |  |  |  |  |
| Low | Reference |  |  |  |
| Medium | 0.87 (0.55, 1.38) | 0.556 |  |  |
| High | 0.40 (0.22, 0.73) | **0.003** |  |  |
| **NK cells activated** |  |  |  |  |
| Low |  |  | Reference |  |
| Medium |  |  | 0.77 (0.51 1.16) | 0.214 |
| High |  |  | 0.55 (0.36, 0.83) | **0.005** |
| **Mast cells activated** |  |  |  |  |
| Low |  |  | Reference |  |
| Medium |  |  | 0.99 (0.62 1.56) | 0.95 |
| High |  |  | 1.86 (1.22, 2.81) | **0.004** |
| **Abbreviations**: OS, overall survival; DFS, disease free survival; HR, hazard ratio; CI, confidence intervals; AJCC, American Joint Committee on Cancer system. | | | | |
